# What's in a name? How organelles of endosymbiotic origin can be distinguished from endosymbionts

**DOI:** 10.15698/mic2019.02.668

**Published:** 2019-01-21

**Authors:** Ansgar Gruber

**Affiliations:** 1 Biology Centre CAS, Institute of Parasitology, České Budějovice, Czech Republic.

**Keywords:** organelle, evolution, endocytobiosis, symbiogenesis, chloroplast, eukaryogenesis, speciation

## Abstract

Mitochondria and plastids evolved from free-living bacteria, but are now considered integral parts of the eukaryotic species in which they live. Therefore, they are implicitly called by the same eukaryotic species name. Historically, mitochondria and plastids were known as “organelles”, even before their bacterial origin became fully established. However, since organelle evolution by endosymbiosis has become an established theory in biology, more and more endosymbiotic systems have been discovered that show various levels of host/symbiont integration. In this context, the distinction between “host/symbiont” and “eukaryote/organelle” systems is currently unclear. The criteria that are commonly considered are genetic integration (via gene transfer from the endosymbiont to the nucleus), cellular integration (synchronization of the cell cycles), and metabolic integration (the mutual dependency of the metabolisms). Here, I suggest that these criteria should be evaluated according to the resulting coupling of genetic recombination between individuals and congruence of effective population sizes, which determines if independent speciation is possible for either of the partners. I would like to call this aspect of integration “sexual symbiont integration”. If the partners lose their independence in speciation, I think that they should be considered one species. The partner who maintains its genetic recombination mechanisms and life cycle should then be the name giving “host”; the other one would be the organelle. Distinguishing between organelles and symbionts according to their sexual symbiont integration is independent of any particular mechanism or structural property of the endosymbiont/host system under investigation.

## INTRODUCTION

“What's in a name? That which we call a rose by any other name would smell as sweet”. These are Juliet's words from Shakespeare's “Romeo and Juliet”, and when I first heard Miroslav Oborník's opinion that mitochondria and plastids should actually be called “bacteria” [1], this is what I thought. Not that bacteria smell particularly sweet, but that, as a scientist, I care much more about what mitochondria, plastids or bacteria *are*, than about how they are *called*. Their names changed throughout the history of biology, but without any doubt, mitochondria, plastids and bacteria have, at all times, retained “that dear perfection”^a^ that makes them attractive to the scientists who investigate them.

Nevertheless, I think that Oborník is right in his statement that there is “no well-defined border between symbiotic bacteria and semiautonomous organelles” [1] and in his insistence on assigning proper terminology to the objects of our research [1]. Semantics are important for science, and not just for science, as the tragic example of Juliet shows.^b^ Calling and treating mitochondria and plastids as “bacteria” would imply that they are the same as bacteria, however, biology has shown on many occasions that they are not. So, instead of smoothing over the differences between bacteria and organelles of endosymbiotic origin, I suggest we work with these differences to refine the definition of organelles, which would allow us to reproducibly distinguish them from bacteria.

## WHAT IS AN ORGANELLE?

The term “organelle” was first introduced as a diminutive analogue to organs of multicellular organisms and applied to diverse subcellular structures observed microscopically in eukaryotic cells. Thus, in the beginning the term had no connection or reference to the evolutionary origin of the structures to which it was applied. However, it is noteworthy that in the case of mitochondria and plastids, their semi-autonomous lifestyles within eukaryotic cells and other similarities to free living bacteria led to hypotheses, already at the time they were discovered, that mitochondria and plastids are related to free-living bacteria [2-6]. The evolution of organelles by endosymbioses remained obscure to mainstream cell biology for a long time, however, Sagan (later named Margulis) brought the topic back to attention in the late 1960s [7] and since then, the evolution of mitochondria and plastids via endosymbiosis has become a well-established theory [2, 5, 8, 9].

Interestingly, since the establishment of this theory there have been discussions to restrict use of the term “organelle” to organelles of endosymbiotic origin only: for instance, Kleinig and Maier exclusively apply the term to mitochondria and plastids in their popular German textbook “Zellbiologie” [10]. This practice of course parts with the original meaning of organelles being “small organs of a cell”. Similarly, Oborník's [1] suggestion to name and consider mitochondria and plastids as bacteria also stresses the distinction between “organelles” and “organelles of endosymbiotic origin”.

## SYSTEMS WITH AMBIGUOUS STATUS

Increased research on protist biodiversity and endosymbiotic systems led to discoveries of endosymbioses at various levels of interdependence and integration of the participating organisms that do not fit easily in the symbiont or organelle categories [2, 11-13]. Examples of such symbioses/evolving organelles include: endosymbiotic bacteria found in cells of insects [14-17]; nitrogen-fixing spheroid bodies found in some diatoms [18, 19]; cyanobacteria-derived chromatophores in the amoeboid Cercozoan *Paulinella chromatophora* [16, 18, 20-22]; zooxanthellae in marine invertebrates [23]; and the various groups of dinoflagellates that contain eukaryotic algae instead of or in addition to the peridinin containing dinoflagellate plastids [13, 24, 25]. In particular, the chromatophores of *Paulinella* are considered examples of organelles “in the making” [26] or organelles that “replay the tape” [27, 28] of plastid evolution. (I do think that the implicitly postulated projection of an evolutionary path based on what is in fact more of a “snapshot” in the evolutionary history of an organism is a bit problematic; however, I agree that the discoveries made in the *Paulinella* system are exciting to a degree that justifies all kinds of interpretations and speculations). For this and many other systems classification as symbiont/organelle/host systems is currently quite unclear and remains a matter of debate (or even taste); this can be seen by comparing the differing views on the status of the *Paulinella* chromatophores presented in [29] and [30].

## WHEN SHOULD A (FORMER) ENDOSYMBIONT BE NAMED AN “ORGANELLE”?

Because they are what they are – small organs of cells – acceptance of their endosymbiotic evolutionary history did not change much in regards to their naming: mitochondria and plastids remained organelles in a “you know it when you see it” kind of way for most scientists. If further distinction was required, gene transfer from the symbiont to the host nucleus, with targeting of the gene products to the organelle, was suggested as the distinguishing feature of organelles by Cavalier-Smith and Lee [31], which has been widely accepted.

Sitte referred to the evolution of organelles from originally independent organisms as “intertaxonic combination” [32-34], the “formation of a stable, and obligatory, endocytobiotic system of taxonomically and ecologically different partners” [33]. Sitte also noted that “it is somewhat difficult to define the new term in an unequivocal way” and that “some criteria that appear useful at first are not really practical” [33]. Sitte went on to discuss that neither coevolution between the partners, nor intracellular lifestyle of the symbiont in the host, an obligatory requirement of symbiosis for the partners, or even the occurrence of horizontal gene transfer between the partners are, on their own, sufficient criteria to make the term applicable to a symbiont/host system [33].

More recently, while commenting on the results of sequencing the genome of the *Paulinella* chromatophores (see original study [35]), Keeling and Archibald [36] discuss three aspects under which the status of an endosymbiont/organelle can be evaluated: (i) genetic integration of the host and endosymbiont, i.e. how many genes are targeted to the candidate organelle and how many have been lost from the endosymbiont; (ii) cytological integration, i.e. how synchronized the partners are in their cell cycles, how endosymbionts are distributed to daughter cells and how stably they are transmitted to the progeny; and (iii) metabolic integration, i.e. how host and endosymbiont metabolisms complement each other. Keeling and Archibald [36] regard all three aspects as providing rather soft criteria for the classification, which does not allow for a definite determination whether a particular endosymbiosis has reached a level of integration that justifies organelle status. They even predict that “drawing a line beneath some and calling them organelles and others endosymbionts will never be completely unambiguous” [36].

Reyes-Prieto *et al*. [14] propose the term “symbionelle” for endosymbionts of insects that have smaller genomes than the theoretically inferred genome size of a “minimal cell” (a cell that could potentially live freely, in a very rich medium that compensates for the reduced metabolic capabilities of this cell, see [14] for details). Symbionelles therefore depend on the help of other endosymbionts or the host for their survival. Reyes-Prieto *et al*. make a distinction between symbionelles and organelles because mitochondria and plastids are surrounded by a double membrane, depend on protein import, are synchronized with the host cell in their divisions, and evolved in unicellular organisms [14].

Nowack [21] notes that many nucleus-encoded proteins that are targeted to endosymbiotic bacteria do not originate from these bacteria: they are either host-derived or the result of horizontal gene transfer from organisms other than the endosymbiont. She therefore concludes that targeting of host-encoded proteins to an endosymbiont might be an early step in the establishment of an endosymbiotic relationship, rather than a late step, and that this targeting might originally allow the host to gain control over the endosymbiont [21]. Only after protein targeting has been established can gene losses from the endosymbiont genome possibly be compensated for by host genome encoded genes, and this would then lead to dependency of the endosymbiont on the host [21]. Nowack suggests, as a good criterion to draw the line between endosymbionts and organelles, the “moment when the endosymbiont – as a consequence of gene loss – becomes dependent for survival and proliferation on the import of nuclear-encoded proteins” [21]. According to Nowack, two more criteria should be fulfilled for organelle status: vertical inheritance and a benefit to the host [21].

McCutcheon and Keeling [37] discuss the symbiont/organelle distinction prompted by the discovery that a nuclear gene of the pea aphid, *Acyrthosiphon pisum*, was acquired at some point from an Alphaproteobacterium and encodes a product that is targeted to a gammaproteobacterial endosymbiont of the aphid (see original study [15]). They ask whether a line between symbionts and organelles truly exists and postulate that if it does in fact exist, it should be “drawn by evolutionary and mechanistic distinctions, not by perceived differences born of tradition, definitions, or historical contingency” [37].

## SEXUAL SYMBIONT INTEGRATION

**Figure 1:**
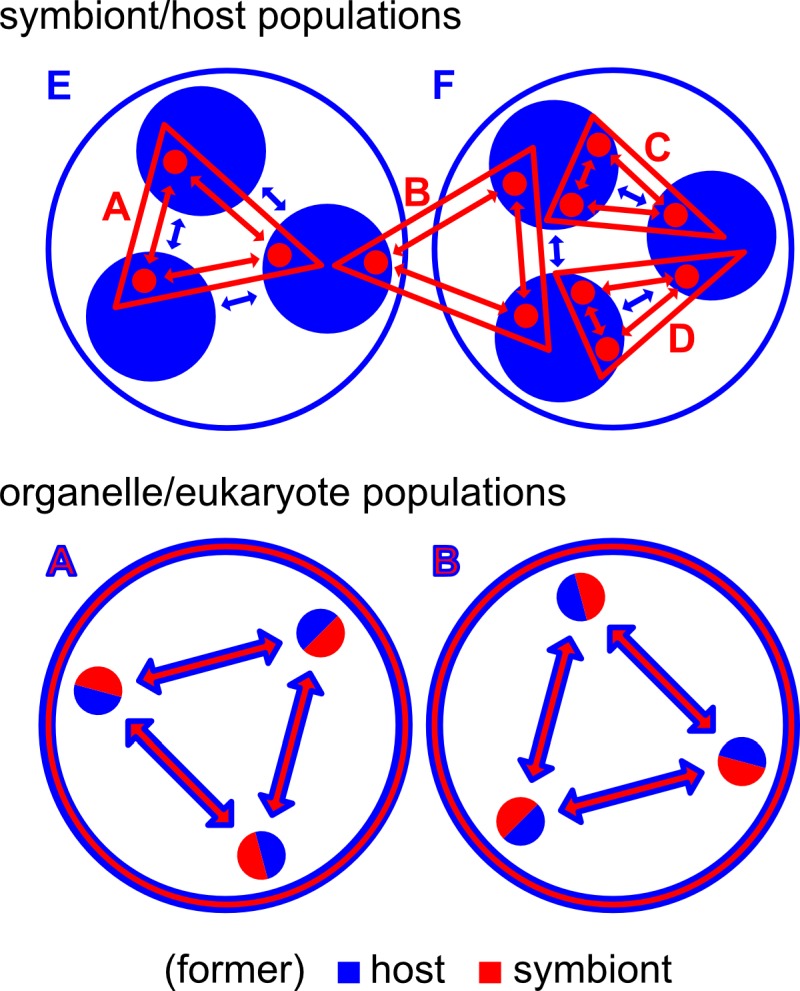
FIGURE 1: Genetic recombination and effective population sizes in symbiont/host and organelle/eukaryote populations. In symbiont/host populations, genetic recombination between individuals of the symbiont and host populations is independent. This allows for host populations to host more than one symbiont population (like symbiont populations B, C and D in host population F), for symbionts to recombine between individuals of different host populations (like symbiont population B which inhabits host populations E and F), and for individuals of different symbiont populations to inhabit the same host individuals. In organelle/eukaryote populations, genetic recombination between individuals of a population is coupled for the former partners, who now have identical effective population sizes; this also means that there is no distinction between individuals of the former host and the former symbiont.

I could not agree more with the above quoted statement by McCutcheon and Keeling, so here is my suggestion for how to draw this line: I think that the key distinguishing feature of organelles is that in organelle/eukaryote systems, genetic recombination between individuals of a population, if it occurs, is coupled between organelle (symbiont-derived) and other (host-derived) components of the cell. This means that the same number of individuals from both former populations recombine (breed), and thus it results in congruence of the former symbiont and host effective population sizes (**Figure 1**).

**Figure 2:**
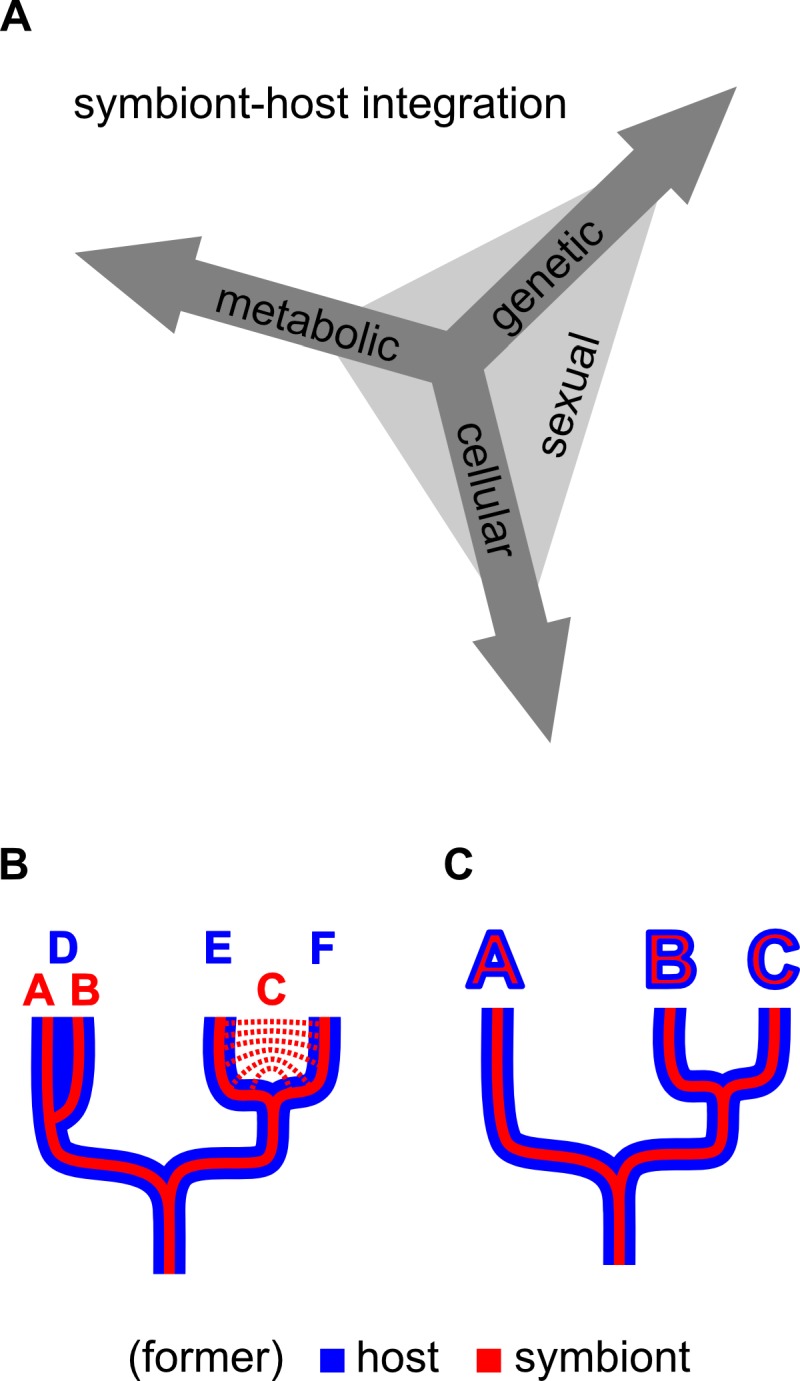
FIGURE 2: Symbiont-host integration. **(A)** Genetic, cellular and metabolic symbiont integration together result in sexual symbiont integration, which can be used to qualitatively distinguish endosymbionts from organelles. **(B)** Host/symbiont system that is not fully sexually integrated; symbiont species A and B evolved in the same host species – D, host species E and F coevolved with the same symbiont species – C. **(C)** Fully sexually integrated host/symbiont system, speciation is coupled between symbiont and host.

I suggest calling this aspect of integration “sexual symbiont integration”: the degree of similarity in heritability mechanisms, effective population sizes, and speciation history (or speciation potential) between the symbiont/organelle and the host (**Figure 2**). The mechanisms that lead to this coupling of recombination are diverse and can result from all three aspects of symbiont integration previously discussed by Keeling and Archibald [36] (genetic, cellular, and metabolic; as explained above) (**Figure 2**).

In the case of genetic integration, gene transfer to the nucleus directly changes the mode of inheritance and recombination of the transferred genes. However, the resulting sexual symbiont integration might be low (if only a few genes are transferred) or asymmetric between a host and an endosymbiont (for instance if the endosymbiont remains viable without the host). Furthermore, there may be physiological advantages of “local” gene expression within an organelle or a symbiont that prevent certain genes from being transferred and thus limit the extent to which an endosymbiont/organelle genome can be reduced [38].

Cellular integration and vertical transmission lead to similar life histories of the partners; however, if the interaction is only obligatory for one of them, sub populations of symbiont or host free-living organisms can arise, and therefore speciation is not strictly coupled between the partners due to some degree of cellular integration alone. Also vertical inheritance of the endosymbiont with respect to the host life cycle does not per se exclude transfer of endosymbionts between individuals of the host or genetic recombination of endosymbionts within the host. Finally, metabolic integration directly leads to obligatory interactions; however, as long as the partners retain their own inheritance and genetic recombination mechanisms, “obligatory” does not mean that the partners are sexually integrated.

While there is some ambiguity or flexibility in how exactly the genetic, cellular and metabolic aspects of integration influence each other and the resulting sexual symbiont integration, I think that, overall, the concept of sexual symbiont integration allows for evaluation of the endosymbiont/organelle/host system according to a qualitative criterion: if the particular combination of genetic, cellular and metabolic integration found in a symbiotic system results in congruence of effective population sizes, genetic recombination, and speciation between the partners, I suggest we call this system fully sexually integrated (**Table 1**). In this case, the endosymbiont should be called an organelle and only one species name (that of the host) should be assigned. The organelles of endosymbiotic origin should then be implicitly called by their eukaryotic species names, as is common practice for mitochondria and plastids.

**TABLE 1.** Criteria for and consequences of sexual symbiont integration.

	**No sexual integration**	**Full sexual integration**
Genetic recombination between individuals	Independent	Coupled
Effective population sizes of partners	Different	Same
Identity of individual	Different	Same
Resulting status	Symbiont/host	Organelle/eukaryote
Resulting taxonomy	Separate taxa	Same taxon

**Figure 3:**
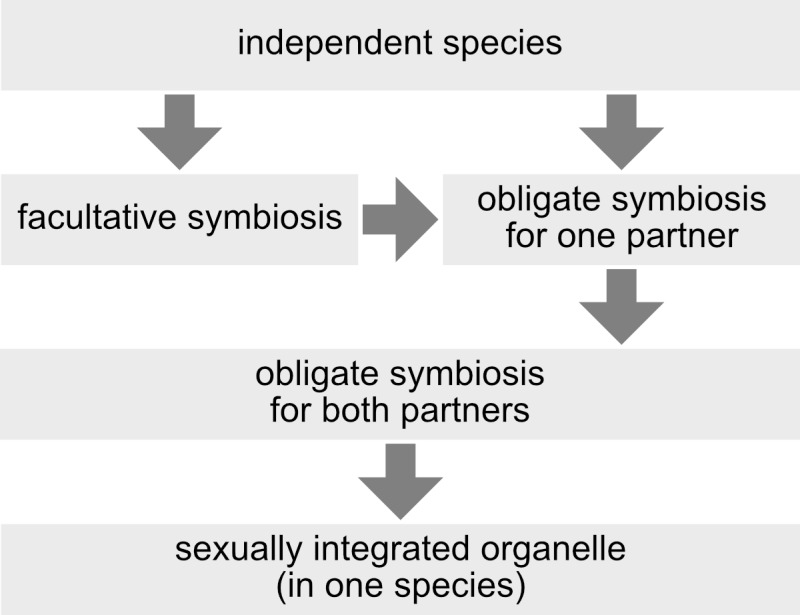
FIGURE 3: Different levels of symbiont-host integration. A symbiosis can start from independent species, or might be obligatory from the beginning, for instance when a symbiont interacts with a new host. In order to become fully sexually integrated, one requisite is that the symbiosis is obligatory for both partners.

This criterion does not rely on any single mechanism (e.g. presence of protein targeting), degree of symbiont reduction (e.g. smaller genome than that of a “minimal cell”), degree of symbiont retention (e.g. has to have a genome), or structural feature with which (endo-) symbiont integration is achieved (e.g. presence or absence of a phagotrophic vacuole), and it is independent of any evaluation of a symbiosis as mutualism, commensalism or parasitism (which is highly subjective and of course depends on one's perspective). Also independent of mechanisms, reduction levels, structures, or winner/looser considerations, three stages between independent organisms and integrated organelles can be distinguished: facultative symbioses, symbioses that are obligatory for one of the partners, and symbioses that are obligatory for both partners (**Figure 3**). Another advantage of the concept of sexual symbiont integration is that horizontal gene transfer to endosymbiont, organelle, or nuclear genomes, which is a prevalent phenomenon [39, 40] of controversial significance [41-43] for organelle evolution, has no direct influence on the sexual integration level of an endosymbiont. The source of horizontally transferred genes, by definition, lies outside of the usually recombining population (or species), so that horizontal gene transfer is always an exception rather than the rule. To further illustrate the concept of sexual symbiont integration, I will discuss two examples: zooxanthellae and mitochondria.

## ZOOXANTHELLAE AND SEXUAL SYMBIONT INTEGRATION

The first example is the zooxanthellae, dinoflagellates (mostly of the genus *Symbiodinium*) that live intracellularily in marine invertebrates (mostly cnidarians). As they are photosynthetic, they render the whole symbiotic system phototrophic [23, 44]. A symbiotic system that is characterized by such close interactions is also termed “holobiont”, a term that was coined by Margulis [45] and that nicely illustrates how the participating organisms depend on their biotic interaction partners in an existential way. Metabolic integration between the symbiont and host as a result of the photosynthetic lifestyle of the holobiont is very high. To complete their life cycle, the symbiosis is obligatory for many of the host species, although for short periods or during certain stages in the lifecycle the host may survive without its symbionts [23, 44, 46]. However, there is an asymmetry in the dependency, as *Symbiodinium* strains can more easily survive without hosts [23, 47]. Genetic integration of the partners in zooxanthellae-containing systems is low: despite complete sequencing of several *Symbiodinium* and coral species genomes [48-50], gene transfer or protein targeting from/to zooxanthellae has not been detected, which means that if it exists, it is most likely not a major contribution to the system. Cellular integration of zooxanthellae is somewhat intermediate: zooxanthellae can be vertically transmitted during sexual reproduction of the hosts; however, in many host species symbionts are freshly acquired in each generation during larval stages [46]. Moreover, host species can be host to various species or strains of zooxanthellae (even within the same individual), as can the zooxanthellae colonize various hosts [23, 44, 51]. Consequently, the effective population size of the symbiont is independent of the population size of the host. Additionally, recombination in host and symbiont populations takes place independently and according to the mechanisms of the respective species. It is therefore conceivable that host or symbiont populations speciate independently, for instance by changing the ranges of possible interaction partners. So in this first example, metabolic integration of the symbiont and host is high, while their genetic and cellular integration is low. Overall, this leads to low sexual symbiont integration.

## MITOCHONDRIA AND SEXUAL SYMBIONT INTEGRATION

The second example I would like to discuss is the mitochondria. Mitochondria evolved from bacteria at the very beginning of the evolution of eukaryotes, and the evolution of mitochondria has even been suggested to mark the prokaryote/eukaryote divide [8, 9]. Their metabolic integration with eukaryotic cells is very high: mitochondrial respiration is the key energy providing reaction in the catabolism of aerobic eukaryotes [5], and also certain anabolic pathways take place in the mitochondria, which means that most groups of anaerobic eukaryotes retain reduced forms of mitochondria [9]. (Only one eukaryote species that has lost its mitochondria is known so far, the the oxymonad *Monocercomonoides* sp. [52].) Genetic integration of mitochondria is also very high: the majority of proteins that are active in the mitochondria are nucleus encoded and the gene products are imported via dedicated protein import systems [8, 9]. Similarly, there is high cellular integration of mitochondria: mitochondria are passed to daughter cells during mitosis and meiosis and are present in eukaryotic cells throughout the cell cycle. Taken together, this means that the sexual symbiont integration of mitochondria is very high. The high degree of genetic integration results in Mendelian inheritance of most of the genes of mitochondrial origin, which means that they also follow the eukaryotic mode of sexual recombination. There is only one type of mitochondria in each eukaryotic individual, and because mitochondria do not participate in the genetic recombination and sexuality that occurs in bacteria, the effective population size of mitochondria is the same as the effective population size of the species to which they belong (somatic cells in multicellular organisms do not count). This also means that mitochondria are subjected to the same mechanisms of speciation as their eukaryote population. Exactly the same? Well, almost, because the mitochondrial genome is an exception to the Mendelian inheritance of chromosomes in eukaryotes. Mitochondria are inherited uniparentally in many species of eukaryotes (although not strictly [53]); however, this still means that mitochondrial propagation to the next generation is coupled to the eukaryotic life cycle of mitosis, meiosis and zygote formation in the same sequence or combination that is followed by the respective eukaryote species, just without the systematic recombination that occurs between eukaryotic chromosomes.

Despite the high level of sexual integration of mitochondria in the eukaryotic cells, co-evolution of the mitochondrial and nuclear genomes persists and the compatibility of mitochondrial genomes with nuclear genes encoding mitochondrial proteins (referred to as cytonuclear compatibility) is important for the fitness of an organism [54-56]. Cytonuclear incompatibility can even lead to reproductive isolation of populations, enabling speciation processes [54-56]. But, also in speciation via cytonuclear incompatibility, the complete eukaryote (including the mitochondria) speciates without separation between the mitochondria and other components of the cell.

It should be noted that there is one challenge to the status of mitochondria as fully sexually integrated organelles, and that is the phenomenon of introgression of mitochondria from one population (or even species) to another (see [55, 57] for explanations and examples). I admit that this process can be viewed as evolution of mitochondria independent from the eukaryote in which they evolved; however, I would like to point out that the introgression of mitochondria into a population of eukaryotes is still linked to the recombination and inheritance mechanisms of the eukaryote species, and that it is, at least initially, coupled to recombination of complete eukaryote haplotypes. Therefore, it is possible that the introgression of mitochondria is accompanied by introgression of the nuclear encoded genes that co-evolved with the introgressing mitochondrial genome [55]. Because the mitochondrial genome itself is small compared to the entirety of gene loci that are mitochondria-derived or that encode proteins that are important for the function of mitochondria, I do not think that the introgression of mitochondria is fundamentally different from the introgression of other gene loci, apart from the obvious difference in inheritance mechanisms (uniparental vs biparental). Interestingly, many phenomena and considerations regarding the sexual integration of mitochondria are also true of plastids; however, in the case of plastids, a more drastic challenge is posed by examples in which plastid genomes are transmitted between plant species via grafts and thus independent of the sexual recombination of the plant species [58, 59].

## RESIDUAL AMBIGUITY IN THE SYMBIONT/ORGANELLE DISTINCTION

In the two examples discussed above, the sexual integration level of the systems can be easily evaluated. The consequences of organelle or symbiont status are quite clear and not in conflict with most existing classifications of the systems. But how does the concept of sexual symbiont integration help evaluate the more controversial endosymbiont/organelle borderline cases? I think that application of the full sexual integration criterion would lead to more instances of organelles in the books, for example in the case of *Paulinella chromatophora* (an obligate symbiosis for both partners and no evidence for sexual recombination in endosymbiont and host). In this case, one species description (the one by Lauterborn [60]) is sufficient. To my knowledge, the chromatophores have not been formally described and I think that such a description is not need.

Regarding the many bacterial symbionts of insects, I would be more careful with assigning organelle status. In vertically transmitted endosymbionts, two categories can be distinguished: obligate (or primary) and facultative (or secondary) endosymbionts [17]. Facultative endosymbionts can be horizontally transferred within and between host species and their genomes show signs of host-independent recombination [17], so facultative endosymbionts are not fully sexually integrated with their hosts. Obligate endosymbionts, however, are not horizontally transferred, their genomes do not show signs of recombination, and they codiversify (speciate) together with their hosts [17]. According to Moran *et al*. [17], obligate endosymbionts “evolved to be dependent on host-based mechanisms for transmission”, and some of them “rival cell organelles in their extent of intimate association on hosts” [17]. Thus, in some cases, they do fulfil the criterion of full sexual integration with their hosts. However, there is one caveat that puts a question mark on their classification as organelles: there are examples of obligate endosymbionts that have been lost from their hosts by replacement with other endosymbionts (see [17] for details). Assigning organelle status to obligate endosymbionts of insects therefore depends on careful investigation of the exact life histories of the endosymbiont and host, as well as on an evaluation of the “risk” that the obligate endosymbiont might be lost by replacement with a facultative one in the future (which, in a certain way, is similar to the replacement risk that mitochondria and plastids are facing, via introgression [55, 57, 58, 59], or via evolutionary plastid replacement [13, 24]). This is obviously a practical challenge that is not entirely favourable to the sexual integration concept. The criterion of protein targeting (as suggested by Cavalier-Smith and Lee [31]), the symbionelle concept (as proposed by Reyes-Prieto *et al*. [14]), or the criterion of genome loss (as suggested by Oborník [1]) are easier to test experimentally than it is to confidentially postulate the absence of independent sexual recombination. (Also, in the *Paulinella* example above I used absence of evidence in my argumentation, but this is of course not strict evidence for absence).

## RESPONSE TO OBORNIK

As mentioned above, I do think that Oborník [1] is right regarding the importance of terminology and also in his emphasis on the distinction between organelles of endosymbiotic origin and other organelles. Admittedly, his suggestion to name and treat mitochondria and plastids as bacteria rather than organelles would eliminate the need for a distinction between symbiont/host and organelle/eukaryote systems. However, this would only be true as long as the endosymbiont retains its genome, because then, according to Oborník [1], the endosymbiont would finally become a “true organelle” (something that I am tempted to nickname an “undead endosymbiont”). One particular advantage of the concept of sexual symbiont integration compared to Oborník's suggestion is that it does not introduce additional taxa or terms. Once an organelle is considered an organelle due to its sexual integration, it can keep whatever name it already had (for instance “chromatophore” or “mitochondrion”), and the nomenclature also does not have to be changed in the event that drastically reduced homologues of this organelle should be discovered (like mitochondria without a genome, so called mitosomes [9]).

## DOMESTICATION AND ORGANELLE EVOLUTION

Metabolic interactions in many cases are the foundation on which symbiotic relationships are established. Furthermore, maintenance of metabolism is a life or death task for all organisms. For these reasons, I think that metabolic integration is a particularly effective way to fix a symbiotic relationship and to make it obligatory for one, both, several, or many partners. In the broadest sense, even the global interplay of organisms with complimentary nutritional modes – for example, oxygenic photosynthesis and aerobic respiration – can be seen as a form of metabolic integration. From this perspective, it appears logical that Margulis, subsequent to her work on organelle evolution, turned to earth science and passionately popularized the controversial Gaia hypothesis [45, 61]. This hypothesis (in brief: earth itself is a self-regulating super-organism that should be considered “alive”, as discussed in all its controversy by Doolittle [62]), although not falsifiable and thus not scientifically useful, is surely thought provoking and probably has helped many scientists to sort out concepts dealing with a plethora of biological or earth science phenomena, such as “regulation”, “control”, or “consciousness” (at least it has made me think about these concepts).

Oborník's [1] comparison of the process of organelle evolution with the domestication of organisms by humans is similarly thought provoking. There is nothing to falsify, but it is inspiring to think about the underlying concepts of dependence, independence, and control. So, how integrated are humans and their domesticated organisms according to the concept of sexual symbiont integration? The answer lies directly in the mechanism of domestication, which is based on intentional choices by humans regarding which individuals of a population of domesticated organisms are allowed to recombine with each other to form the next generation, and continued selection of the progeny (for many generations) according to certain desired criteria. This means that, while in the “care” of humans, the domesticated species has lost its independence regarding recombination and population size development, which is the prime criterion for sexual symbiont integration. Interestingly, in the case of organelle evolution, sexual symbiont integration usually requires synchronisation of the life cycles of the host and endosymbiont. This is not the case in the domestication of organisms by humans. The human “host” effectively adjusts the applied selection practices to the life cycles and generation times of the candidate/target organism and these are as diverse as they possibly can be (dog, chicken, wheat, potato, and yeast are just a few examples). Does this mean that our domesticated organisms are fully sexually integrated with *Homo sapiens*? I do not think so. There are two important differences between domesticated organisms and fully integrated organelles, and these differences are not in size or in the fact that our domesticated organisms are not endosymbionts^c^.

One difference between domesticated organisms and fully integrated organelles is the facultative nature that is retained in the relationship between humans and their domesticated organisms. We, the humans of this world, have always domesticated other species. In fact, I think that our habit to intentionally cultivate and domesticate other species is the most exclusive feature among the distinguishing features of humans (in comparison to other animals) like language, self-recognition, tool utilization and development, cultural heritage, and so on. But even though it appears impossible or at least very painful to imagine the current human population on earth without the contributions of staple food like wheat, rice or potatoes, historically there have always been human populations who have thrived without one or another, or even without many of the highly useful domesticated organisms. So for humans, dependence on the domesticated organisms is not strict: we need domesticated organisms to improve our lives, but we can do fine without any particular one. (Yes, even the end of cats would not mean the end of humanity.) Likewise, in most cases, the domesticated organisms are not strictly dependent on their relationship to humans. This is proven by the countless feral populations of formerly domesticated organisms around the world. (Cat-lovers, if it helps: the end of humanity would most probably not mean the end of cats.)

The other important difference between domesticated organisms and fully integrated organelles is the independence of speciation. Due to the nature of the domestication process, it is quite difficult to assign species status to domesticated organisms compared to their wild counterparts; however, there are clear examples of speciation in the hand of human breeders, for example the allopolyploid speciations that occurred during the domestication of hexaploid wheat [63]. This demonstrates that speciation of domesticated organisms is not coupled to speciation of the breeders.

## HOW MANY MICROBIOMES?

Following Oborník's suggestion [1] would also increase the recognition of our bacterial heritage and, at the same time, highlight the more recently evolved dependencies of many eukaryotes on bacteria. In classifying mitochondria as bacteria, humans (and many other organisms) would be understood to contain even more bacteria than they do according to the classical view [1]. To further classify our microbiome, Oborník suggests we distinguish “extracellular” from “intracellular” microbiomes and include mitochondria in the latter [1]. I agree that the extracellular/intracellular distinction is highly relevant, in particular because intracellular bacteria are often highly dangerous pathogens [64], while our extracellular microbiome is mostly beneficial [65]. However, I do not think that mitochondria should be considered a part of our microbiome. Of course, it is important to consider their bacterial nature, when developing medical treatments with antibiotics, for instance. But as explained above, human mitochondria are human (or any other organism's mitochondria are a part of that other organism).

We inherited our mitochondria from our mother even before our zygote had formed, and when we die, our mitochondria also die. The only way for our mitochondria to make it into the next generation is to follow the life cycle of their human (the maternal inheritance is coupled to sexual reproduction, so there is no mitochondrial propagation without human reproduction/recombination). This is in stark contrast to our bacterial or eukaryotic microbiome: the composition of our microbiome can change throughout our life time [66], the species that are part of our microbiome can be transmitted between individuals [67], and when we die, the bacteria that made up our microbiome can still have their bacterial ways with us [68].

In my opinion, there is a qualitative difference between mitochondria and the bacteria that interact with us. The extent to which mitochondria are integrated into eukaryotic reproduction and recombination processes (their sexual integration) does not justify classifying them as independent organisms. This would also not do justice to the many intracellular bacteria that, despite high integration levels, are not sexually integrated with their hosts in a way that leads to congruent evolutionary trajectories.

## WHO IS THE HOST?

Oborník also brought up the fascinating idea that if our perspective were changed from the “eukaryotic view” to the “bacterial point of view” of endosymbiosis, “humans (and other eukaryotes) would just be very sophisticated motile incubators for bacteria”. He went on to remark that humans might have a hard time admitting that “some “primitive” prokaryotic cell enslaved an ancestor of eukaryotes and that we are only here to provide a safe space to our bacterial lords” [1]. I agree, modesty is not a particular strength of us humans, however desirable improved awareness of our bacterial heritage and support system might be. I am no exception: after having accepted the challenge and having enjoyed the “bacterial view” on endosymbiosis, I still think that, despite the dependence of eukaryotes on their organelles of endosymbiotic origin, there is an asymmetry in the relationship that allows for distinguishing between the host and the symbiont. Bacteria are part of our (or of all eukaryotes') heritage and, by having become organelles, they persist in the vast majority of eukaryotes. This indeed enabled the assimilated bacteria (read “organelles”) to exist in habitats and under conditions in which bacteria alone could not exist. But the genetic mechanisms and evolutionary processes that led to the diversification of eukaryotes are, after all, eukaryotic. Going back to the distinction between a fully sexually integrated organelle and a host/endosymbiont system, I think that it is the partner who maintained its life and cell cycles through the process of symbiont integration that should be considered the “host” and deserves to be the name giving partner.

Finally, I would like to return to Sitte's concept of intertaxonic combination [33], which emphasizes that during organelle evolution two species unite into one. This also means that there is one species less than in the beginning. Schenk therefore saw Sitte's intertaxonic combination as an “antisymbiotic process”, which leads to the “dissolution of the ‘symbiotic relation’” [69]. According to Schenk [69] this process is asymmetrical: the host gains genetic information, while the symbiont is reduced until, what Schenk calls “inequal intertaxonic combination” [69, 70] is completed. Applying the criteria of sexual symbiont integration will help us to recognize whether this has already happened in many of the recently discovered or yet to be discovered, “candidate organelles”.

## CONCLUSION

General acceptance of the evolution of mitochondria and plastids by endosymbiosis and the discovery of endosymbiont/host systems that show properties that were previously thought to be unique to organelles have resulted in the need for a better defined border between endosymbiont/host systems (which require independent species names) and fully integrated organelles (of one eukaryotic species). To make this distinction, I suggest we evaluate the degree of sexual symbiont integration, which results from genetic, cellular, and metabolic integration. If the effective population sizes, access to genetic recombination partners, and speciation history (or potential) are the same between the partners, the host and symbiont should be considered one species, and this species should be named after the partner who has maintained its life cycle and genetic recombination mechanisms.

## ENDNOTES

a Just paraphrasing Juliet; of course, I know that mitochondria, plastids and bacteria, like everything in biology, are far from being perfect.

b Juliet's word play regarding the rose might appear harmless, but the following (mutual) denial of her and her lover's names soon leads to the untimely loss of her and her lover's lives. “Loss” is a euphemism, but elaborating more would be off topic.

c Not yet; but there is hope that our list of domesticated organisms might include endosymbionts in the future.
